# 1897: A Retrospect

**Published:** 1898-01-01

**Authors:** 


### 1897: A Retrospect.

THE year of Jubilee has come to an end—the year in which people of every class have vied with one another in celebrating the Record Reign of our gracious Queen has vanished into the limbo of the past; and now, on the very first day of the opening year—for does not our title page bear the date of New Year's Day?—we take the opportunity of wishing to our readers all health, happiness, and prosperity. Every year has characteristics of its own, and no one can turn over the publications, the books, the periodicals, and the newspapers of the year 1897 without perceiving that its whole literature has been tinged with an air of retrospect. Whatever the subject of the hour, writers have striven with one accord to compare the present with the past; the title "1837—1897" has been made a prominent headline in relation with every conceivable topic; and it must be confessed that many people have become rather tired of retrospect in every shape and form. Still, as years roll by, and as one after another they take their place in history, it is well, even if only as an exercise in self-examination, to look over the events of the past; and so it happens that, full as the year has been of retrospects, we commence the new one with yet another—a Retrospect of 1897.

### The Hospital.

And first we must say a word in regard to the continued success and prosperity, and the constantly-enlarging circulation of THE HOSPITAL—a success which should be gratifying to its readers, its editors, and its proprietors alike. As to the latter we need say nothing, the matter is one which goes without saying; as to our readers, they may feel assured that the more prosperous the paper the greater will be the return which they will get for their subscription; and as to ourselves, while accepting with due gratitude the compliment which is implied in an increasing circulation, we may express the hope that it also means an appreciation and an approval of the step which was taken a little more than a year ago in arranging for a joint editorship, with the object of keeping THE HOSPITAL more fully in touch with the actualities of medical science.

THE HOSPITAL is now produced under the joint editorship of Sir Henry Burdett and Dr. Solomon Smith, with the assistance of a considerable staff of helpers in the various departments of knowledge which are dealt with every week, and we have every reason to believe that this method of collaboration has proved useful to the paper and acceptable to its readers.

### The Prince of Wales's Hospital Fund.

One of the most important events of the year, so far as hospitals are concerned, has been the establishment of the Prince of Wales's Hospital Fund. His Royal Highness, in initiating this Fund, hoped not only to do something to relieve the incubus of debt which seemed in many cases to hamper the operations of the hospitals, but to draw into the ranks of annual subscribers whole classes that had hitherto stood entirely aloof. How far the latter object has been gained remains to be seen. The test will come when the time for repeating the subscription arrives. But as a means of collecting a noble sum for the benefit of the hospitals of the metropolis the Fund has been an unmitigated success, nearly £230,000 having been subscribed.

Naturally the hospital world has been somewhat exercised over the question, "What will he do with it?" And, indeed, the disposal of such a sum, especially if in any large degree it should become an annual contribution, is a very serious matter. The Prince of Wales, however, has shown himself to be very clear-sighted in regard to economic and social questions; he has good advisers, and we are very confident that this Fund will be so distributed as not merely to give to the hospitals so much cash in hand, but to exercise a good influence in regard to the management of the hospitals of London for a long time to come.

### Hospital Reform.

But little progress has been made on this subject, and such as is apparent can hardly be said to have sprung from the "reformers." The Hospital Reform Association has made some investigations into the working of special hospitals, but otherwise, beyond drawing attention to certain sides of the question, its efforts do not seem to have been productive of great results.

On January 25th a special meeting of the Charity Organisation Society was held, at which a committee was appointed to promote the establishment of a Central Hospital Board for London, and a further meeting was held on December 13th. In the meantime a less formal association of hospital managers has been formed, with the object of securing some degree of uniformity of method at the larger hospitals.

If, then, we bear in mind that those who have the responsibility of distributing the Prince of Wales's Fund, the Sunday Fund, and the Saturday Fund are able to exercise considerable influence in favour of any scheme which may seem to them, either individually or collectively, to be of importance, it is obvious that the machinery for encouraging reform is plentiful, if not redundant. Curiously enough there seems as yet to be but little unanimity as to the character or amount of the "abuse" which takes place, while there is still less in regard to the guise in which "reform" should come; so that, amid such a multitude of counsellers and so great a variety of cooks, there seems considerable danger of the broth being spoiled. We should have thought that, whatever else was uncertain, this at least was clear, that in presence of the alleged abuse of hospitals by people who are said to be well able to pay, the very first essential in any scheme, whatever direction it may afterwards take, should be the submission of every application for hospital relief to the scrutiny of a skilled inquiry officer. But no, the reformers will have none of him; the *British Medical Journal* holds him up to scorn, and Mr. Loch asks instead for an "almoner," a "person skilled in the method of charity, who could give good and ingenious advice"—as if any hospital out-patient would go to an almoner who had nothing but advice to offer, so long as the hospital doors were open for him to go in and get his physic without any inquiry. We have maintained that an inquiry officer is the first step in any reform, and, so far as we can see, the only real advance that has been made during the year on the subject of hospital reform is the resolution of the Hospital Sunday Fund to the effect that the existence of a special and efficient inquiry officer, for the detection of abuse in the out-patient department of a hospital, shall be an important factor in determining the merits of that hospital and the amount of money which shall be allotted to it. This ought to mean much, and it is at least something. The rest is talk—interesting, but sterile.

THE GENERAL MEDICAL COUNCIL.

The May meeting of the Council was presided over by the President, Sir Richard Quain, notwithstanding that he was only just recovering from serious illness. In November, however, the chair was taken by Sir William Turner. Great regret was expressed in the Council, and we may add was felt throughout the profession, at the absence of one who had for so long attended with unremitting diligence to the affairs of the General Medical Council. Among the events of the year must be noted the action of Dr. Rentoul, one of the newly-elected direct representatives, who surprised his friends, and outdid all that his critics had ventured to suggest, by suddenly throwing up his seat.

However advantageous to the Council as a deliberative and judicial authority his action in so doing might be, there can be no doubt that it was a great blow and no small discouragement to those who pinned their faith upon direct representation. The profession, however, was quick to see the humour of the situation, and saved itself from ridicule by replacing him by a strong man in the person of Professor Victor Horsley. In addition to the usual deliberations upon penal cases, the chief points to be noted in regard to the year's work of the Council are the announcement that the new edition of the Pharmacopœia is practically completed; the adoption by the Council of the report of the Education Committee, which will have the effect of weeding out some of the weaker examinations in general education, and thus checking the incursion of half-educated youths into the profession; and lastly, the issue of a notice by the Council in regard to the employment of unqualified assistants—a notice which, if consistently acted upon, must have the effect of very materially altering the conditions under which practice is carried on in many parts of the country. In spirit we are with the Council in this matter, although we cannot but wish that the affair had been taken up in a somewhat more businesslike manner. What is quite clear is that if this notice is to be a dummy, a mere *brutum fulmen*, it will act with great injustice, causing enormous inconvenience to all just men while letting the sinners continue their evil practices unchecked. On the other hand, if it is to be sternly enforced, the Council ought to have been much more careful and explanatory in their definitions.

TYPHOID FEVER.

The four chief epidemics of the year—those namely at Maidstone, Lynn, Clifton, and Belfast—are of such recent date that we need here only refer to them. It has been frequently said that the recent outbreak at Maidstone is the most extensive epidemic of typhoid fever that has ever occurred in England. This, however, is an error. Neither in total number of attacks, nor in the proportion of the population affected, does it come up to the epidemic which occurred at Over Darwen in the year 1874, when, out of a population of about 22,000 persons, 2,035 were attacked within a very short period, and of these 104 died. In this case the proof of the fouling of the water supply was very clear. The patient to whom the mischief was traced, having contracted the disease elsewhere, came home ill and died in a house some little distance from the town. The drain into which the excreta of this patient were thrown crossed the line of the water main, and from sinking of the land, or some other cause, a free communication had been established between them, with the result that the contents of the drain were sucked freely, indeed regularly, into the water main. So does history repeat itself! The same may be said of other epidemics. A little research into the history of the past would soon discover almost a replica of each, and one cannot but feel that the time has come when, considering the extent and the accuracy of the knowledge which we possess in regard to the etiology of this disease, something very much more definite should be done to limit its diffusion than has so far been attempted.

In view of the powers possessed by the sanitary authorities in regard to the trade in meat and milk, and, so far as adulteration is concerned, in regard to all trades dealing with articles of food, it seems absurd, and, indeed, it is absurd in the highest degree, that those who trade in drinking water should be allowed to occupy such an independent position as they do. It is now quite clearly recognised that if the water supplies of our towns are to be safe for drinking purposes they must either be derived from sources which are entirely free from all chance of contamination, or they must be carefully and thoroughly subjected to certain processes which are known to be capable of rendering them pure and fit to drink. Yet with this knowledge staring us in the face, we hand over the business of collecting and supplying this necessary of life to commercial companies, whose object is the earning of dividends. We set up monopolies, and voluntarily deprive ourselves of the right of competing with them; we allow these companies to collect water from impure or doubtful sources, and to use such methods of purification as they think fit, and having done this we do not even insist that our inspectors shall have the right of free access to the works; we impose no penalty for the distribution of typhoid-poisoned water, and, after thorough English fashion, we content ourselves with a good growl and a public subscription for the sufferers when a great epidemic arises. The lesson of the year is that this matter is far too important to be allowed to go on in its present happy-go-lucky fashion, for England is becoming more crowded every year, and pure water is becoming more difficult to find.

ANÆSTHETICS.

The art of producing anæsthesia has arrived at such perfection that the discussions which have taken place on the subject have not been much concerned with questions of their efficiency, but have revolved continuously around the problem how to avoid the dangers which accompany their use. That in the aggregate these dangers are very considerable no one can doubt, and the reports which we have published from time to time of deaths under anæsthetics have helped to draw attention to the importance of the subject.

Among the many communications on anæsthesia which have been contributed to the various journals, none probably have excited so much interest as that by Mr. Leonard Hill, in which he showed how direct and immediate was the action of chloroform on the heart, and how important, in regard to the occurrence of sudden death, was its power of paralysing the mechanism by which compensation for alteration of position is secured. As to the fact that under the influence of chloroform the blood pressure is lowered all are agreed; the question is as to how this lowering of the pressure is brought about. Mr. Hill considers that from the first moment of its administration chloroform produces a gradually increasing paralysis of the heart, while at the same time it abolishes the tonic contraction of the peripheral blood-vessels. The result is that the circulation fails, and that, as an incident in this failure, the respiratory centre is no longer supplied with blood, and respiration ceases. He maintains, however, that the cessation of respiration is a result of circulatory failure and of the action of the anæsthetic upon the heart and the peripheral vascular system. The position taken up by Mr. Leonard Hill has been somewhat violently assailed, but we think that he has well held his own in the controversy which has arisen, and his theories have this in their favour, that they have led to certain very definite teachings as to the prevention and treatment of accidents from chloroform. As regards prevention, clearly the great thing is to avoid any sudden overcharge of chloroform in the pulmonary blood, and with this object to administer the drug with regularity and in measured doses; and, as regards treatment, to be ready with artificial respiration so as to tide the patient over the crisis until the heart can become charged with less poisonous blood. It must be definitely remembered that inversion and pressure on the abdomen are worse than useless if the heart is already full, as is almost sure to be the case if syncope occur, as it often does, when the patient after struggling and holding his breath at last inspires a mass of over-charged air. In such cases if artificial respiration, performed in the horizontal position by forcibly compressing the chest rhythmically, does not at once produce an effect, it should be continued in the vertical feet-downward position, and under no such circumstances must the abdomen be compressed or invertion practised.

In considering the year's work in regard to anæsthetics we cannot but feel that there is but little hope of any real advance on the clinical side until some form of collective investigation is organised. Deaths from anæsthetics must of necessity be rare in any one man's practice even at the worst, and no one can fairly dogmatise as to the safety of this or that method until he has used it in a very large number of cases. It is, in fact, a question of using the same method in thousands of cases, and such a question can only be decided by a collective inquiry carried out on identical lines at a considerable number of the larger hospitals. If such an investigation were properly organised, it might, even in a single year, at least decide something. At present, notwithstanding all that is being done, nothing is or can be decided, and we have to fall back on the physiologists, or else on general impressions.

### The State of the Army in India.

During the past year attention has been drawn very strongly to the prevalence of venereal disease among our troops in India. It is no new thing. Every year the presentation of the army medical report gives occasion for a few articles in the press upon this unsavory subject. Usually these complaints have fallen on stony ground, but by some good fortune they this time got listened to and bore good fruit. There is nothing like a practical example, and when it came out that of the 5,822 men detached for field service with the Chitral Relief Force, 462, or nearly 8 per cent., had to be rejected for this group of diseases contracted before crossing the frontier, and 270, or an additional 4½ per cent., had to be transferred from the field hospitals to the base for the same cause, even the British public made up its mind to discuss the subject. The most striking and curious thing that came out was that coincidentally with this great increase in venereal disease there had been a marked diminution in drunkenness and crime in the army. It seemed, in fact, as if the prevalence of the vices which lead to these diseases had more to do with opportunity than with any innate immorality among our young soldiers.

It was urged by those who had officially investigated the matter that greatly increased powers should be granted to the authorities for preventing the access of infected persons to the cantonments, and that methods of treatment analogous to those in operation for other infectious diseases should be adopted; and, after considerable discussion a Bill was passed in July giving the necessary powers.

It will be interesting to observe what happens. Putting all questions of morality on one side, it is clear that English soldiers in India are far too expensive articles to be placed at the mercy of a lot of dirty native women, and that some very definite restrictions must be placed upon what is in fact a trade which is dangerous to the efficiency of our army. At the same time, we cannot hold Mr. Thomas Atkins as entirely free from blame when he does what he must know is likely to render him incapable of fulfilling his side of the bargain made with the recruiting sergeant. Certainly some form of penalty should fall upon those who so betray their trust. Probably, when the proposed improvements in the conditions of service come into operation, our soldiers will be less ready to do things that may lead to their discharge than they seem to be at present.

### The Plague.

When the year opened, the outbreak of plague in Bombay was in full swing. It was quite impossible at the time to arrive at any true estimate of the extent of the mortality, so numerous were the cases which were either hidden or wrongly notified, but it certainly was very large. The epidemic began in September, 1896, and made comparatively little headway until the beginning of December, when it rapidly assumed grave proportions. Not only were large numbers of deaths from plague registered every day, but the general mortality increased enormously, and it was impossible to avoid the conclusion that a large proportion of this increase was due to deaths which really ought to have been put down to plague. The attempts of the Bombay Muncipality to deal with the epidemic led to nothing but discord and failure, the disease was found to be spreading to other parts of India, and at the beginning of February it became clear to the Central Government that much stronger measures must be applied if the disease was to be brought under control. The result was the passing of the Dangerous Epidemic Disease Bill, which gave almost unlimited powers to deal with the disease in a radical manner, included powers to enter, cleanse, whitewash, &c., all houses; to destroy rubbish; to remove plague cases to isolation hospitals; and to do many other things from which the municipality had been held back by ideas about caste, and by respect for religious and racial customs. And it was high time that some action should be taken, for it now turns out from the report of the committee appointed to carry out the Act, that from August up to the beginning of March 19,849 persons had died from plague. The plague had prevailed in Bombay for six months, and yet so terribly had it been allowed to get ahead of the provisions for controlling it, that when the committee was constituted only two public hospitals for plague patients were in existence, and even in these there were no nurses. The total accommodation at that time seems to have been for less than 300 patients, while from 900 to 1,000 plague deaths were occurring weekly. The city, however, was at once divided into 10 administrative districts, and 15 Government hospitals were organised, besides those provided by various castes for their own members, the military gave great help, medical assistance and instruction was provided, nurses were called up, the sick were attended to, segregation and quarantine camps were organised, and an enormous amount of sanitary work was done. Quickly the epidemic abated; but even now it has not ceased, and the question still remains unsolved how far the active interference of the Government has been influential in staying the plague. It would be extremely gratifying to feel that we had got the whip hand of this disease; but although it is true that in April—that is, not much more than a month after the committee got to work—the prevalence of the disease had greatly diminished, it is also true that from observation of the natural history of plague in other places it had been again and again foretold, even during the height of the epidemic, that such would be the case; and, unfortunately, even yet, and with the very much smaller number of cases to deal with, it has not been found possible to stamp out the disease. There can be no doubt that the ultimate result of the epidemic in regard to the sanitary condition of the city will be good; but it will not do to be too confident as to the efficacy of segregation and quarantine as means of controlling epidemic plague. Compared with some other epidemics, the death-rate in this outbreak appears to have been but small. It has, however, caused a very serious loss of life, the deaths from plague in the Bombay Presidency having already amounted to nearly 50,000.

We may safely say that one of the great lessons that the plague in India has taught us is that the time has not yet come, or nearly come, when any large amount of power of self-government can be safely placed in native hands. It is disappointing, especially to those who have looked forward to a speedy extension of local self-government in India. But to those who have watched Indian affairs most closely it is by no means surprising. England must still supply the Rulers of India.

### Serum Diagnosis.

The utility of this method of diagnosis in typhoid fever has been fairly established, and it has been largely brought into practical operation during the year. No doubt the interpretation of the reaction is in many cases far from certain, and is complicated by the facts that the reaction itself is always a matter of degree, and that its occurrence is influenced by past as well as by present illness; still, it is an additional and very useful means of diagnosis in doubtful cases, and has proved of considerable utility in giving a clue to medical officers of health. As seems to be inevitable in these matters, the Americans were before us in taking official cognisance of the new method, and rendering it available for use by the profession; but one of the most striking instances of its applicability is to be found in the recent outbreak of milk typhoid in Clifton, where the medical officer of health, suspecting that some recent cases of "influenza" might really be cases of enteric, volunteered to examine the blood by Widal's test. Seventy-six specimens of blood were examined, and most of them gave a positive reaction. The development of the epidemic quickly showed it to be one of typhoid fever of a virulent type.

### Serum Treatment.

The treatment of disease by the injection of serum obtained from animals rendered immune by the repeated injection of the toxins, and even of the actual microbes of the disease in question, has well held its ground in regard to diphtheria, the report of the medical officers of the Asylums Board Hospitals being again of a distinctly favourable character. Many attempts have been made in the course of the year to produce serums which shall act in the same way in regard to other diseases, but so far the results have been much less definite than those obtained in the treatment of diphtheria. Among other diseases experimented on in this direction we may mention tetanus, leprosy, relapsing fever, pneumonia, puerperal fever, erysipelas, pyæmia, surgical tuberculosis, adenitis, phlyctenlar ophthalmia, &c. The anti-streptococci and the antipneumonic sera seem to be the most hopeful of those which have this year been under investigation. Unfortunately it would appear that the diseases in which these organisms occur are very apt to be, or soon to become, mixed infections, and thus are less amenable to such treatment than otherwise perhaps they might be. Another form of so-called "serum" medication has been tried in cases of toxæmia. This, however, is rather what we should call transfusion of saline fluid. The *modus operandi* is doubtless by dilution of the toxins. As showing how wide a bearing modern conceptions of the dependence of various morbid states upon the presence of toxins have, we would mention that Tommasoli has used this "dilution method," as we should prefer to call it, in cases of severe burns. It seems to be now generally admitted that some very toxic substances are thrown into the circulation when large areas of skin are severely burned, and two cases are reported by Tommasoli in which he injected saline solutions (chloride and bicarbonate of sodium) apparently with good effect.

### Diphtheria and Infant Schools.

Amid the general fall in the death-rate from zymotic diseases the steady increase in the mortality from diphtheria becomes all the more striking. Whereas in times gone by diphtheria used to be a disease of the country, its incidence now falls chiefly upon the towns; and at the present time the loss of child life from this cause in London is so great as to become a matter of the most serious public concern.

A point to which we have again and again drawn attention is the effect of what has come to be called "school influence" on the prevalence of diphtheria. The School Board officer is a potent factor in other matters besides those pertaining to mere education, and it is impossible to avoid the conclusion that the steadily increasing pressure which is being brought to bear upon parents to send their children to school at the very age when they are the most susceptible to infection—to schools in which they are crowded together quite irrespective of the conditions of the homes from which they come—has a great deal to do with the really alarming increase which has of late years taken place in the prevalence of diphtheria in certain portions of the metropolis.

In view of the now well ascertained fact that the infection of diphtheria is present in the throats of many children who exhibit no manifestations of the disease, it seems inconceivable that so infective a malady should not be increased along with increasing opportunities of personal infection, and it is much to be regretted that this view of the matter should not be fully recognised by those who are responsible in the matter. If the infection takes place within the schools, a stricter discipline and an improved system of ventilation ought to lessen the evil; while if it takes place in the playgrounds, and during that short but dangerous period of childish intercourse which intervenes between dismissal in the school-rooms and dispersal in the streets, it ought not to be beyond the powers of a great organisation like the School Board to prevent any such intercourse taking place. In any case, the School Board is entrusted with compulsory powers in regard to education, and practically has a free hand in regard to money, and the least that one can expect is that, whether the education given is good or bad, in any case it shall be so given as not to interfere with the health of the children, and not to entail upon them the risk of catching so grave a malady as diphtheria. The present death-rate from diphtheria is too big a price to pay for the more or less problematical benefits of Board School education as applied to infants.

### The Children of the State—and Others.

An event which is likely to prove of considerable importance to London is the issue of an order by the Local Government Board, directing that the Metropolitan Asylums Board should take charge of the following classes of Poor Law children—(*a*) Those suffering from ophthalmia and other contagious diseases of the eye, and from contagious diseases of the skin and scalp; (*b*) those requiring special treatment during convalescence, or the benefit of seaside air; (*c*) those who, by reason of defect of intellect or physical infirmity, cannot properly be trained in association with children in ordinary schools; and (*d*) those who are sent to a workhouse on remand by the magistrates.

Needless to say, this is a much smaller measure than was hoped for by many, while it is a much larger one than is considered justifiable by some. The Metropolitan Asylums Board have already taken steps to provide ophthalmic schools and seaside convalescent homes, and in a little time we shall doubtless see whether the benefits which are being thus provided for paupers can be kept to the pauper class alone. The work of the Asylums Board has always fallen into two very distinct groups—the treatment of the sick and the care and training of the imbecile; and now to these is added the combined treatment and education of certain chronic sufferers. Already the Managers have thrown open their hospitals for acute diseases to the whole population, whether paupers or not, and we think that ratepayers will somewhat anxiously inquire how long it will be before they also throw open to all and sundry these new institutions and these seaside homes. Ophthalmia and ringworm and slow convalescence are terrible trials to hundreds of children just above the pauper class, and the difficulty of educating defective children falls far more heavily on the respectable poor than it does on those for whose maintenance the guardians are responsible. Undoubtedly a very strong claim could be made out for the admission of such children to the institutions which are going to be managed by the Asylums Board, a body which, so far as its hospitals are concerned, has already cut itself adrift from pauperism; and it will be interesting to see, when this claim is made, as it surely will be, how long it will be resisted. The year 1897 has seen the introduction of what may turn out to be a nice little end to a nice little socialistic wedge in regard to the rearing of the defective children of the poor. Many questions, both in sociology and heredity, are likely to be touched thereby.

### New Honours.

In a year of Jubilee, honours were sure to be distributed. Space prevents our giving a full list of those who have been selected for recognition. One honour, however, must be mentioned, both because it is a new thing to find a medical man in the House of Peers, and because the recipient of such high honour from the Crown is the very man to whom the medical profession delights to see honour done. That Lord Lister should be the first medical peer is a matter which is pleasing to all who care for the scientific side of medicine, and to all who recognise how directly life-saving is true science; and it is also peculiarly gratifying that the highest honour hitherto given to any medical man should have fallen upon one whose influence upon modern medicine and surgery is so widely recognised and so highly appreciated throughout the world.

Among the new medical baronets are Sir Samuel Willis, Sir Richard Douglas-Powell, Sir William MacCormac, Sir Thomas Smith, and Sir James Reid.

New knights are: Sir W. R. Gowers, Sir Felix Semon, Sir George Duffey, Sir William Thomson, and Sir Richard Kynsey. Among those who take the title by virtue of the Order of the Bath we note the names of Sir Henry C. Burdett and Sir Richard T. Thorne, but space forbids our filling up the list. The honours bestowed are many, and they are well deserved.

